# Safety Study and Compositional Analysis of the Svarnvir-IV Tablet With Special Reference to Its Therapeutic Utility in SARS-CoV-2

**DOI:** 10.7759/cureus.75438

**Published:** 2024-12-10

**Authors:** K. Ramachandra Reddy, Priya Kumari, K. Vinay, Jyotsna Singh, Brijesh S Chauhan, Prabhat Kumar, Saripella Srikrishna, Deepak Kumar, Madhumita Mishra, Muniyandi Singaravel, N. K Prasad, N S Anuraag, Chetan Sahni, Sanchit Sharma

**Affiliations:** 1 Department of Rasa Shastra (Ayurvedic Pharmaceutics) Faculty of Ayurveda, Institute of Medical Sciences, Banaras Hindu University, Varanasi, IND; 2 College of Medicine, Bharti Vidyapeeth Deemed University (BVDU) Medical College, Sangli, IND; 3 Department of Biochemistry, Faculty of Science, Banaras Hindu University, Varanasi, IND; 4 Department of Zoology, Faculty of Science, Banaras Hindu University, Varanasi, IND; 5 Department of Metallurgical Engineering, Indian Institute of Technology, Banaras Hindu University, Varanasi, IND; 6 Department of Anatomy, All India Institute of Medical Sciences, Gorakhpur, Gorakhpur, IND; 7 School of Pharmaceutical Education and Research, Jamia Hamdard, New Delhi, IND; 8 Department of Research and Development, Aimil Pharmaceuticals India Limited, New Delhi, IND

**Keywords:** ayurveda, cell-based assays, drosophila, elemental composition, sars-cov-2, svarnvir tablet

## Abstract

Aim

Traditional Ayurvedic herbo-mineral medicines have proven their potential in managing COVID-19. Cell-based assays of the Svarnvir-IV tablet demonstrated the virucidal activity against SARS-CoV-2 and its therapeutic action, along with safety in cytotoxicity, has been proved. In the present study, in vivo, safety profile and compositional analysis of the Svarnvir-IV tablet were performed.

Methods

The safety and potency of the Svarnvir tablet were evaluated comprehensively through in vivo drug screening on Drosophila, along with elemental composition analysis of Svarnvir tablets using atomic absorption spectroscopy (AAS), inductively coupled plasma-mass spectroscopy (ICP-MS), X-ray diffraction (XRD), and scanning electron microscopy energy dispersive spectroscopy (SEM-EDS).

Results

The Svarnvir tablet was found safe in Drosophila and their larvae up to the dosage of 1 mg/ml. In comparison to the control, morphologically and physiologically healthy and active flies were observed without any change in circadian locomotor activity rhythms or activity patterns. In addition, the elemental composition of Svarnvir tablets was evaluated using AAS, ICP-MS, and SEM-EDS, and the microstructure was examined by means of XRD and SEM.

Conclusions

Overall, these findings will contribute to an accessible and safe therapeutic approach for traditional age-old Ayurvedic medication to combat SARS-CoV-2 variants.

## Introduction

SARS-CoV-2 infections, which led to the COVID-19 pandemic, continue to impart a global burden on the health infrastructure. Coronavirus outbreaks in the past and present have infected humans and animals with respiratory, intestinal, hepatic, and neurological disorders [1,2]. Many variants of SARS-CoV-2 with novel spike protein mutations have emerged over the past few months, affecting the clinical as well as epidemiological aspects of the COVID-19 pandemic [3,4]. The appearance of the SARS-CoV-2 variant of concern, like the Delta variant, over the preceding few months has been correlated with its high R0 value as well as its capability to escape from the immune response of vaccines and past infections [5,6]. Global policymakers, like the World Health Organization (WHO), were putting in continuous efforts to curtail the infection rates for the protection of vulnerable immune-compromised populations, as we are witnessing emerging new infections [7,8]. Many known as well as newer allopathic drugs were introduced against the coronavirus activity in vitro. However, scientific investigation of antiviral formulations from the Indian traditional system, i.e., Ayurveda, is yet to be explored.

Based on the clinical features of COVID-19 as per Ayurveda, SARS-CoV-2 infection may be compared with Ayurvedic disease pathology as one of the varieties of Sannipata Jwara associated with cough wherein three Doshas/genomics, viz. Vata, Pitta, and Kapha get vitiated. Currently, there is no proof that the contemporary medical system can prevent or treat the illness, but the time-tested traditional wisdom of Ayurveda can aid in disease management and prevention [9,10]. Several Ayurvedic medical formulations like Swarna Bhasma and Rajata Bhasma have been reported as efficient treatments against COVID-19 due to their capacity to lower inflammatory markers in plasma like interleukins, interferons, and TNF levels [11]. Combinational therapy of a nutraceutical; resveratrol and zinc were also found effective against COVID-19 [12]. Indian traditional medicinal plants like Withania somnifera, Phyllanthus emblica, Glycyrrhiza glabra, Tinospora cordifolia, Asparagus racemosus, Ocimum sanctum, and Azadirachta indica have the power to boost our immunity, providing an opportunity for discovering new and effective chemical moieties against viral targets [13,14]. These herbal medications can be beneficial as low-cost treatments of choice for the treatment or prevention of coronavirus [15]. The upgraded acumen for Ayurvedic medications and treatment has explored the knowledge of the interaction of viruses and hosts, which helped in the understanding of the pathway linked with the therapeutics of these Ayurvedic antiviral regimens.

The antiviral and virucidal (irreversible) activity of an Ayurvedic herbo-mineral formulation, i.e., the Svarnvir-IV tablet (450 mg), has been selected, already assessed, and found to have potential as a formulated agent against SARS-CoV-2 infection [16]. The in vitro study evaluated the Svarnvir-IV tablet-450 mg for its potent protease inhibitor activity associated with antiviral activity against SARS-CoV-2. It is found that the Svarnvir-IV tablet is an inhibitor of the causative virus (SARS-CoV-2) based on the above results. Svarnvir-IV tablet, when incubated with SARS-CoV-2 virus at 0.1 multiplicity of infection (MoI) for two hours, exhibited virucidal activity against SARS-CoV-2 with an EC50 value of 0.0058 mg/ml. It also exhibited therapeutic activity when treated with cells infected with the SARS-CoV-2 virus (0.1 MoI) for one hour, two hours, and four hours post-infection, with EC50 values of 0.094 mg/ml, 0.023 mg/ml, and 0.05 mg/ml, respectively. The original supporting data obtained from this study, along with existing Ayurvedic traditional information, will help to understand the potential of the Svarnvir-IV tablet in the treatment of COVID-19 [16].

The tablet designed and formulated by the first author as per Ayurveda principles contains both extracts from medicinal plants and active mineral particles. The major components include Guduchi satva (water-soluble extract prepared from the stem of Tinospora cordifolia), incinerated mineral micropowders, i.e., Yashada bhasma (incinerated zinc nanosized particles), Svarna bhasma (incinerated gold nanosized particles), and Loha bhasma (iron oxide nanoparticles). The leaf juice of Vasa (Adhatoda vasica) is used for the trituration of all ingredients for making tablets.

## Materials and methods

Svarnavir tablet-450 mg

**Figure 1:**
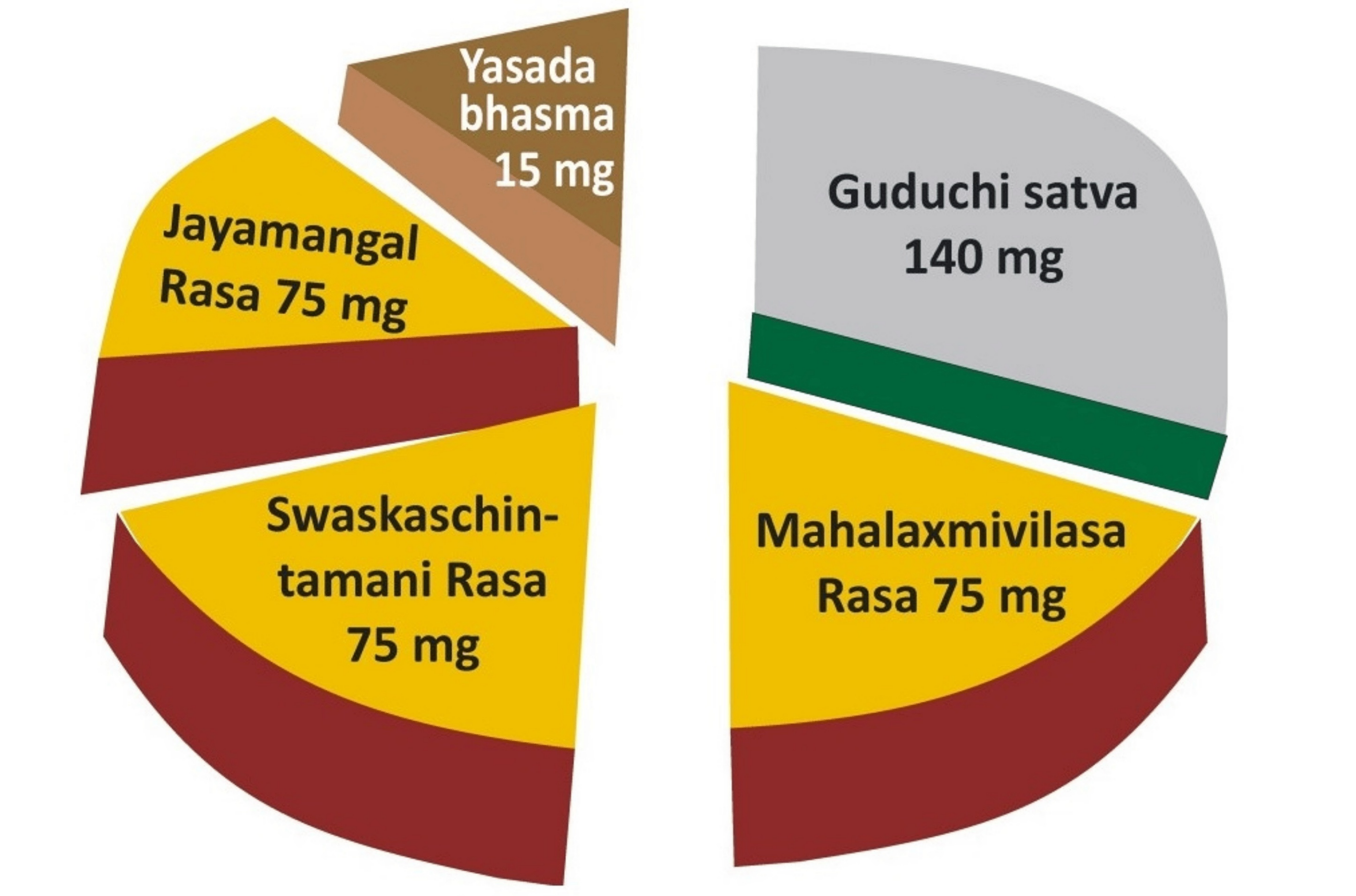
Ratio of ingredients of the Svarnvir-IV tablet

The study was conducted at Banaras Hindu University and ITC Ltd. (India). In the present study to evaluate the toxicity effects of the Svarnvir tablet, a cell viability assay, a biochemical assay, and a locomotor activity rhythm assay were conducted. For elemental and micro-structural analysis of the Svarnvir tablet, three spectroscopic techniques were used, i.e., inductively coupled plasma mass spectroscopy (ICP-MS), atomic absorption spectroscopy (AAS), and scanning electron microscopy in energy-dispersive spectroscopy (SEM-EDS). Additionally, the compound formed from the elements present in the Svarnvir tablet (Figure 1) was determined with the help of X-ray diffraction analysis.

Fly strains and culture conditions

Wild-type (OregonR+) fly strains were obtained from the Bloomington Drosophila Stock Centre, Indiana, USA, and used in this study. All crosses were carried out at 24±1oC in a biological oxygen demand (BOD) incubator in vials containing standard corn-meal agar media.

In vivo toxicity study in Drosophila model

In Vivo Drug Screening

In order to determine the toxicity effects of Svarnvir tablet, the wild-type flies were treated with different doses of Svarnvir tablet from lower to higher concentration, i.e., 0.05 mg/ml, 0.1 mg/ml, 0.5 mg/ml, and 1 mg/ml, during various developmental stages starting from embryo to adults. All the developmental stages (embryo, larva, pupa, and fly) were observed to evaluate the toxicity effects of the Svarnvir tablet.

Cell Viability Assay

The cell viability assays were performed in wild-type parental flies, and their F1 progeny were treated with Svarnvir tablets. The drug-treated parental flies and wandering third-instar larvae of the F1 generation were subjected to an MTT assay to examine the cell viability. In each group, a total of 15 parental flies and L3 larvae treated with different concentrations (0.05 mg/ml, 0.1 mg/ml, 0.5 mg/ml, and 1 mg/ml) were separately processed for the cell viability assay as per methods described by Chauhan BS et al., 2022 [17]. All experiments were performed in triplicate.

Biochemical Assays

Biochemical assays were done in tissues treated with 0.05 mg/ml and 1 mg/ml of Svarnvir tablets of wild-type parental flies, wandering third instar larvae, and adult flies of F1 progeny. In each group, a total number of 15 flies and larvae (N=15) were considered for the hydrogen peroxide (H2O2), superoxide dismutase (SOD), and lipid peroxidase (LPO) activity assays using the methods described by Chauhan BS et al., 2022 [17]. All experiments were performed in triplicate for the study.

Locomotor Activity Rhythm Assay

To determine the effects of the Svarnvir tablet on the locomotor activity rhythm and activity pattern of the wild-type strain of Oregon R+Drosophila, four groups (N=70; 14/group) of the newly emerged sexually naïve male and female flies were used. The flies were anesthetized and loaded in individual 5 mm Pyrex glass tubes/activity tubes, with one end filled with standard fly food formulated with different doses of Svarnvir tablets, i.e., 0.05, 0.1, 0.5, and 1.0 mg/ml, and an opposite end covered with a cotton plug. The activity tube was placed in the DAM2 activity monitor (Trikinetics Inc., MA, USA), which was housed in the incubator. A 12:12 h light/dark (LD) schedule with a constant temperature (25 0.1°C) was maintained in the incubator. The data recording was done using the software DAMSystem311 (Trikinetics Inc., MA, USA) at a six-minute (0.1 h) bin length [18,19] and data processing through DAMFileScan113 (Trikinetics Inc, MA, USA) for further analysis [20]. The preparation of the activity record (actogram), time-series analysis (activity counts/min), and activity profile (average activity count/min) were done using Clock Lab, version 2.63 (Actimetrics, USA). Flies that did not meet the minimum duration of recording were eliminated from the analysis. The statistical analysis was done by using Microsoft Excel 2019 (Microsoft Corporation, Redmond, Washington, USA), and Google Sheets (Google LLC, Mountain View, California, USA) were used for box plot preparation. One-way analysis of variance (ANOVA) with Tukey’s multiple comparisons test was performed using GraphPad Prism, version 8.4.4 (679) (Insight Venture Management, LLC, NY, USA) for Windows (Microsoft Corporation, Washington, USA).

Elemental and Micro-Structural Study

The sample (Svarnvir tablet) was crushed manually using a pestle and mortar to subject it to further structural, microstructural, and elemental characterizations. To analyze the elemental composition of the powdered sample, the energy dispersive spectroscopy (EDS) attachment with Scanning Electron Microscope (Model: EVO-Scanning Electron Microscope MA15/18. Company: Carl Zeiss Microscopy Ltd., Jena, Germany, EDS: 51N1000-EDS System) was utilized. Individual elemental composition and elemental mapping of the sample were carried out in the SEM's secondary electron (SE) mode. However, for better accuracy of composition and trace metals present in the sample, atomic absorption spectrophotometry (AAS) and inductively coupled plasma-mass spectroscopy (ICP-MS) were performed. Calibration standards for both spectroscopic techniques were obtained from Sigma Aldrich (Merck KGaA, Darmstadt, Germany).

AAS analysis was executed using an Elico Ltd. (Elico-Connecting Science and Lab, Hyderabad, India), atomic absorption spectrophotometer (SL 168). The powdered samples of known weight were digested in aqua regia and were stirred for 30 minutes. The digested samples were diluted to 10x, 100x, and 1000x using distilled water. Samples in distilled water were sent into a pneumatic nebulizer at a feed rate of 6.0 mL min-1 by a Teflon capillary tube and a high-density polyethylene (HDPE) spray chamber. Acetylene was passed (at 18 psi pressure) as a fuel along with dry air (continuous 45 psi pressure) as an oxidant to produce a flame. The flow rates of fuel and oxidant were maintained at 3 and 2 L min-1, respectively. 

 ICP-MS analysis was carried out using a Thermo-Fisher Scientific (Thermo Fisher Scientific Inc., Waltham, MA, USA) inductively coupled plasma mass spectrometer (iCAPRQ). To examine the phases formed by the existing elements in the sample, X-ray diffraction (XRD) was carried out on the powder. The Rigakuu Miniflex 600 Desktop X-Ray diffraction system (Rigaku Analytical Devices, MA, USA) is was utilized for the purpose, with Copper K-alpha radiation with a wavelength of 1.5406 Å. The scan range was 20-60 degrees with a scan rate of 5º per minute and a step size of 0.02.

## Results

Antiviral assessment for Svarnvir tablet against SARS-CoV-2 virus

The Drosophila model safety study has been calculated based on the clinical study protocol for the human dose of Svarnvir tablet-450mg oral administration twice in a day.

Effect of Svarnvir tablet on developmental stages of OregonR+

**Figure 2:**
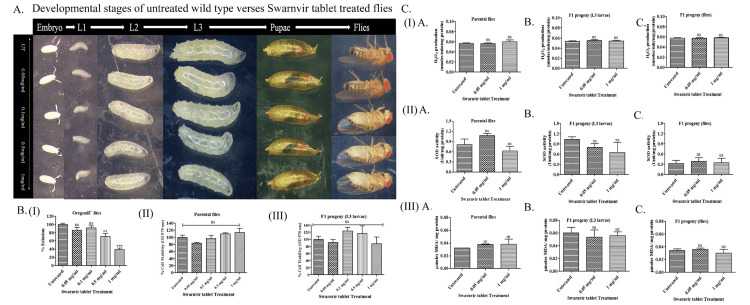
Comparative analysis of developmental stages, viability, and oxidative stress in Drosophila F1 generation treated with Svarnvir tablet A. F1 generation showing the comparative status of various developmental stages between untreated and Svarnvir tablet-treated fly groups. B. Histogram showing the effect of Svarnvir tablet on relative percentage eclosion of F1 progeny (I). B. Cell viability analysis of wild-type parental (II) and its F1 progeny 3rd in star larvae (III) after administration with Svarnvir tablet. C. Oxidative stress analysis includes hydrogen peroxide (I), superoxide dismutase (II), and lipid peroxidase (III) assays of wild-type parental flies, third instar larvae, and adult flies on the administration of Svarnvir tablet. The data is ascribed as non-significant (ns, p > 0.05) and significant (**p<0.001, ***p<0.0001) using one-way ANOVA.

The toxic effects of the Svarnvir tablet were examined on different developmental stages of wild-type, starting from embryos to adult flies. The untreated wild-type flies and their progeny were considered as respective control groups. The F1 generation of untreated flies was considered as 100% (N=185) and flies treated with Svarnvir tablet doses of 0.05 and 0.1 mg/ml showed 86% and 98% flies eclosion, respectively. The Svarnvir tablet-treated flies with 0.05 and 0.1 mg/ml concentrations exhibited normal and healthy development at all developmental stages, although there was a slight reduction in the number of flies eclosed as compared to the untreated group. However, the eclosed flies were healthy as their control counterparts. The 0.5 and 1 mg/mL concentrations of Svarnvir drug-treated flies showed 71% and 39% fly eclosion (Figures 2A, 2B (I)), respectively. The reduced percentage of fly eclosion could be due to less egg laying, as all the larvae, pupae, and adult flies that emerged out of these concentrations are healthy as control group flies.

Effects of Svarnvir tablet on cell viability

The cell viability analysis was performed in wild-type parental flies, and their F1 progeny was treated with Svarnvir tablet dosages ranging from 0.05, 0.1, 0.5, and 1 mg/ml. The untreated parental flies and their progeny exhibited 100% cell viability. Svarnvir tablet-treated parental flies showed 84%, 97%, 135%, and 114% improvement in cell viability on dosage ranges of 0.05, 0.1, 0.5, and 1 mg/ml, respectively, whereas at similar dose concentrations, third instar larvae showed 90.7%, 123.3%, 115.8%, and 87.7% improvement in cell viability in contrast to untreated wild type. Here, Svarnvir tablet concentrations of 0.05, 0.1, 0.5, and 1 mg/ml improve the metabolic functions by enhancing the cell viability of parental flies and their third instar larval progeny (Figure 2B (II and III)). The overall study revealed that the administered dosage of Svarnvir tablet to parental flies and its progeny statistically shows non-significant changes in cell viability.

Effects of Svarnvir tablet on oxidative stress assessment

To evaluate the oxidative stress levels using hydrogen peroxide (H2O2), superoxide dismutase (SOD), and lipid peroxidase (LPO) assays in untreated and Svarnvir tablet-treated wild-type parental flies, their third instar larvae and adult progeny were examined. Here, two dosages of Svarnvir tablet, i.e., 0.05 and 1 mg/ml, were designated for the biochemical analysis (Figure 2C (I, II, and III)). In the case of parental flies, H2O2 production of untreated flies was equivalent to flies treated with Svarnvir tablets at 0.05 mg/ml and 1 mg/ml (Figure 2C (IA)). The H2O2 production in F1 progeny larvae and adult progeny has also similar consequences as compared to untreated ones (Figure 2C (IB,IC)). Similarly, SOD activity was also measured, and it was found that 0.05 mg/ml treated parental flies exhibited 1.33-fold SOD increased activity, while 1 mg/ml treated parental flies demonstrated 1.30-fold reduced SOD activity in contrast to untreated wild-type flies (Figure 2C (IIA)). The SOD activity of Svarnvir tablet at a dose of 0.05 mg/ml treated F1 larvae manifested gradually decreased 1.3-fold in contrast to 1 mg/ml treated F1 larvae shown 1.6-fold higher SOD activity compared with untreated wild-type (Figure 2C (IIB)), but in adult progenies, there were not many alterations seen in SOD activity (1.2-fold in 0.05 mg/ml and 1.1-fold in 1 mg/ml) (Figure 2C (IIIC)). Moreover, malondialdehyde (MDA) levels were also measured as a by-product of lipid peroxidation in parental flies, their third instar larvae, and their F1 adult progeny (Figure 2C (IIIA,B,C)). The MDA level in 0.05 mg/ml Svarnvir-treated parental flies was 1.18-fold slightly higher than in 1 mg/ml treated parental flies, in which the MDA level is one-fold versus untreated wild type (Figure 2C (IIIA)). However, Svarnvir tablet treated F1 larvae and their adult progeny having equivalent levels of MDA production (1.1-fold) in contrast to untreated (Figure 2C (IIIB,C)). Thus, the overall study revealed that there were no significant alterations observed in oxidative stress of Svarnvir tablet-treated parental flies, their F1 third instar larvae, and adult flies in comparison to untreated wild type.

Effects of Svarnvir tablet on locomotor activity rhythm

**Figure 3:**
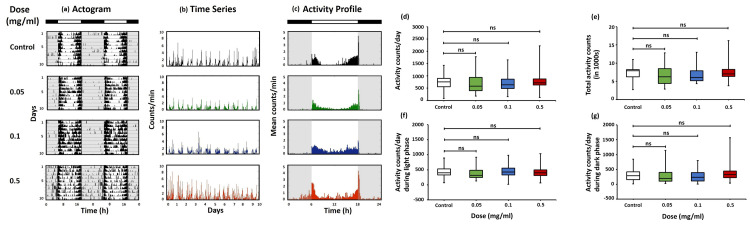
Locomotor activity analysis of control and Svarnvir tablet-fed Drosophila Representative locomotor activity records of control and doses of Svarnvir tablet-fed group flies. (a) Representative double-plotted actograms of control or untreated, 0.05, 0.1, 0.5, and 1.0 mg/ml concentrations of Svarnvir-fed flies. A black and white horizontal bar indicates a 12:12 h light-dark cycle. The black vertical bar indicates activity bouts, the grey background indicates lights off, and the white background indicates lights on. (b) Representative time series (counts/min) recording of control and different doses of Svarnvir-fed flies. (c) Representative activity profile (mean counts/min) recording of control and different doses of Svarnvir-fed flies. A black and white bar indicates a 12:12 h light-dark cycle. The vertical bar indicates activity bouts, the grey background indicates lights off, and the white background indicates lights on. Box plots of activity counts per day (d), total activity counts (e), activity counts/day during the light phase (f), and activity counts/day during the dark phase (g) of the control and different doses of Svarnvir-fed Drosophila groups. The boundaries of the box indicate the 25th and 75th percentiles, the horizontal line marks the median, and the whiskers extend 1.5 times the interquartile range from the 25th and 75th percentiles. The ‘ns’ indicates a symbol for not significant. Statistical analysis shows no significant difference between control and Svarnvir-fed groups (p>0.05), (N=70; 14 per group).

The effect of different doses of the Svarnvir tablet on the circadian rhythm of the wild-type strain of OregonR+ was studied. The activity records (actogram) of flies fed with different doses of Svarnvir tablets (0.05, 0.1, 0.5, and 1.0 mg/ml) display entrainment, i.e., the daily onset of activity synchronized with the lights on. The Drosophila showed a bimodal activity pattern, one in the morning and the other in the evening (Figure 3A, 3C). It also showed an anticipatory rise in locomotor activity in light and dark (LD) cycles just before the change from dark to light and from light to dark. The actogram did not show any variation in the onset of activity, duration of the activity, or bimodal activity pattern of Svarnvir-fed group flies compared to the control. However, the 1.0 mg dose-fed group shows a little more anticipatory activity and a statistical significance of p<0.05 (Figure 3F), but this doesn’t affect survival. The phase angle difference during the onset of activity as well as the offset of activity is unaffected by the different doses (Figure 3A). The activity counts of Drosophila for a corresponding number of days are shown in the time series graph (Figure 3B), and the activity profile graph did not show any significant changes when compared to the control (Figure 3C). The activity profile of respective doses displays activity count (α) during the light phase (active period) and some activity bouts (ρ) during the dark phase (rest period). When compared to the untreated group, the activity profile at different doses does not demonstrate a significant difference.

Effects of Svarnvir tablets on the activity counts

The daily activity counts of different doses of Svarnvir-fed group flies (N=70; 14/group) did not show any significant changes when compared to the control (p>0.05) (Figure 3D). Though the mean activity counts in the 0.05 mg/ml and 0.1 mg/ml fed groups showed slightly lower than the control group. Also, the evaluation of total activity counts did not show any discernible difference between the Svarnvir-fed group and the control group (p>0.05) (Figure 3E). Similarly, the activity counts of the Drosophila during the light or active phase (Figure 3F) of the LD cycle did show significant changes in the Svarnvir-fed group as compared to the control group (p<0.05) but did not affect the survival. The activity count in the rest period, i.e., during the dark phase (Figure 3G), did not show any significant changes when compared to the control group (p>0.05).

Elemental and structural analysis of Svarnvir tablet

The randomly shaped powder particles of the sample can be viewed clearly in the SEM. Contrast differences observed between different sets of particles could be due to the differences in the chemical compositions. Those particles with a relatively brighter appearance are less conducting and thus show a charge build-up, while those with grey contrast are comparatively more conducting. EDS analysis of the particles confirms the approximate chemical composition of the sample.

The weight percentages and elemental composition of the Svarnvir tablet were analyzed using various techniques, including EDS-SEM, X-ray diffraction (XRD), atomic absorption spectroscopy (AAS), and inductively coupled plasma mass spectrometry (ICP-MS), depending on the availability of corresponding standards.

*EDS-SEM Analysis* The elemental composition of the tablet was evaluated using energy-dispersive X-ray spectroscopy combined with scanning electron microscopy (EDS-SEM). The weight percentages of individual elements identified in the sample were determined.

*Elemental Mapping* Elemental mapping conducted via EDS-SEM provided spatial distribution data for key elements within the powdered sample. This analysis included mapping of oxygen (O), sulfur (S), calcium (Ca), lead (Pb), iron (Fe), gold (Au), zinc (Zn), arsenic (As), copper (Cu), and silver (Ag). The maps revealed that Ca, Fe, S, and O were the dominant elements, with detectable levels of Au, Ag, Pb, Cu, Zn, and As. This approach offered an indirect measure of element uniformity across the sample and their relative concentrations.

*Scanning Electron Microscopy and EDS Spectrum* Secondary electron (SE) images of the powder sample were obtained at magnifications of 2000× and 5000×, providing detailed insights into the microstructural features of the sample. The EDS spectrum confirmed the presence of multiple elements within the analyzed region, supporting the quantitative data from the elemental mapping.

*X-ray Diffraction Analysis* X-ray diffraction patterns of the powdered Svarnvir tablet identified calcium zinc carbonate and sulfur as the predominant phases, as indicated by their relative peak intensities. Additionally, iron oxide phases were detected, and the presence of elemental gold (Au) was confirmed based on characteristic peaks in the diffraction pattern.

*AAS and ICP-MS Analysis* Further elemental composition analysis of the Svarnvir tablet was performed using AAS and ICP-MS. These techniques provided quantitative data on the elemental constituents, complementing the results obtained through EDS-SEM and XRD.

Together, these analytical approaches offer a comprehensive understanding of the elemental composition and structural characteristics of the Svarnvir tablet, providing valuable insights into its material properties.

## Discussion

**Figure 4:**
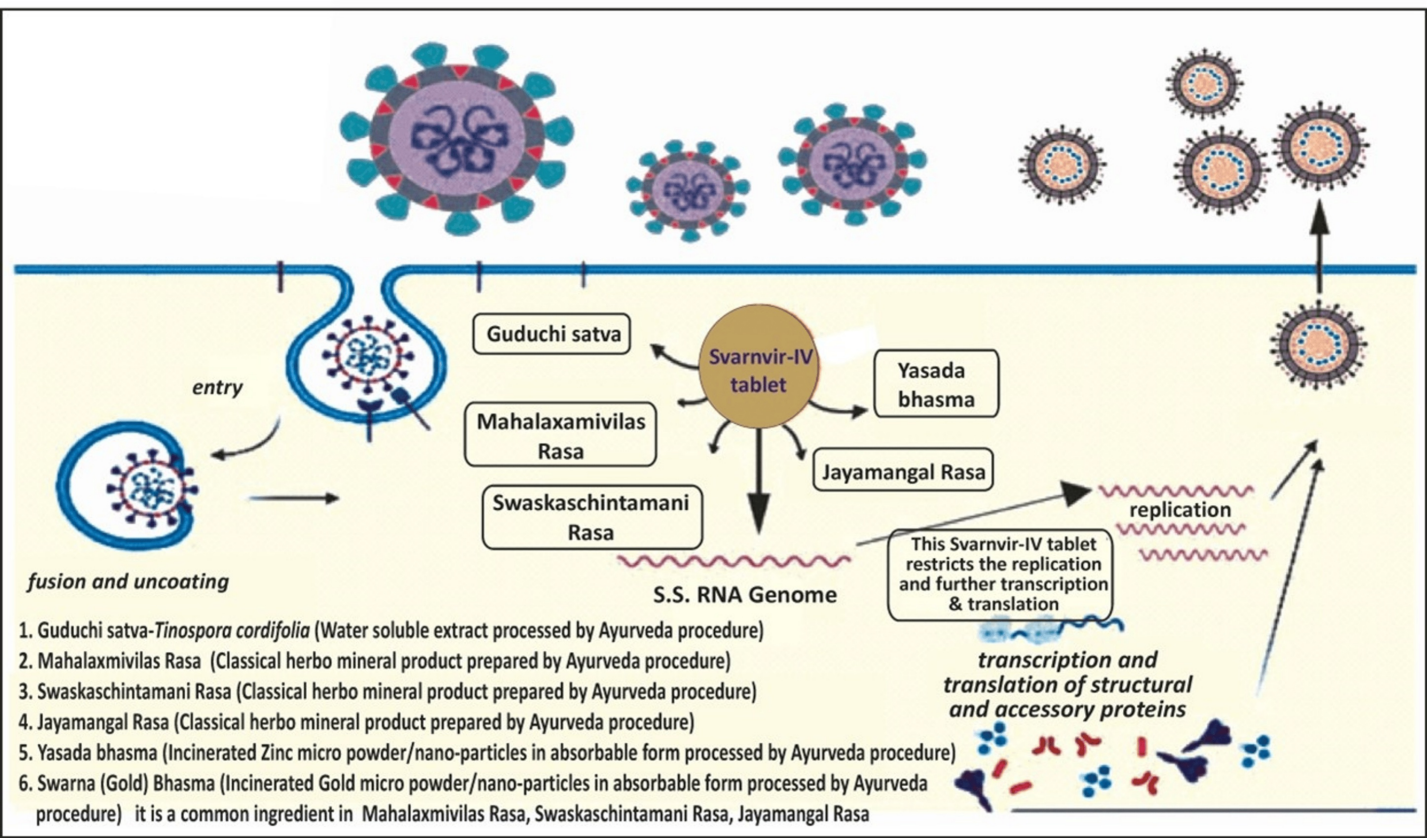
Schematic diagram showing Svarnvir-IV tablet composition and probable action on SARS-CoV-2 virus Image credit: Author Reddy

Across the globe, scientists are investigating potential treatment options for COVID-19 from Ayurvedic and herbal medicinal systems of healthcare. The Indian traditional Ayurveda system of medicine includes a broad aspect of immunity by its rejuvenation therapy Rasayana [21,22]. Traditional uses and scientific evidence on a number of medicinal herbs and their phytochemicals have indicated that these plants can serve as a novel source of natural products against many lethal viral infections [23], with a special consideration on COVID-19 [24]. The composition and probable mechanism of action of the Svarnvir-IV tablet on the SARS-CoV-2 virus are illustrated in Figure 4.

Guduchi satva extracted from T. cordifolia, which is an important constituent of Svarnvir tablets, contains several phytochemical constituents/metabolites [25,26,27]. Tinocordiside, found in Guduchi, has been shown to inhibit the viral protease in a molecular docking study [28]. A study revealed the potential of T. cordifolia constituents to inhibit the SARS-CoV-2 S protein from binding to the ACE2 protein receptor in human host cells. T. cordifolia has also been found to decrease the primary SARS-CoV-2 protease Mpro or 3Clpro [28]. SEM-EDS, ICP-MS, and XRD analyses confirmed the presence of gold particles in the Svarnvir tablet. As per the crystallite size evaluation from XRD analysis using Scherrer’s equation, it was found that the Swarna bhasma (incinerated gold nanoparticles) is around 30-60 nm; however, it is also supported by the XRD and tTransmission electron microscopy (TEM) results mentioned in the previous reports [11]. The Svarnvir tablet contains absorbable gold nanoparticles that prevent viral entry, adhesion, and multiplication during their early stages by entering cells [29]. The sublingual administration of gold bhasma also showed maximum bioavailability [30]. The effectiveness of gold nanoparticles as a virucidal agent is widely known [31]. Auranofin, a rheumatoid arthritis medication licensed by the US Food and Drug Administration (FDA) that contains gold and triethyl phosphine, effectively reduced viral replication and its inflammatory cascade in human lung cells. By increasing the generation of endogenous protective factors, gold nanoparticles also alleviate the cytokine storm [29]. Also, recent studies explored the possibility of gold nanoparticles as a vaccine adjuvant against SARS-CoV-2, due to the strong response of gold-adjuvanted antigen against the virus in BALB/c mice [32]. It is well established that during coronavirus infection, endoplasmic reticulum (ER) stress and unfolded protein response (UPR) activation play a crucial role in viral replication and pathogenesis [33]. Infection with SARS-CoV-2 increases the expression of the ER protein folding chaperones GRP78, GRP94, and other ER stress-related genes to maintain protein folding [34]. Thus, it was stated that auranofin could affect the protein synthesis machinery of the SARS-CoV-2 by inhibiting the redox enzymes such as thioredoxin reductase [29].

**Figure 5:**
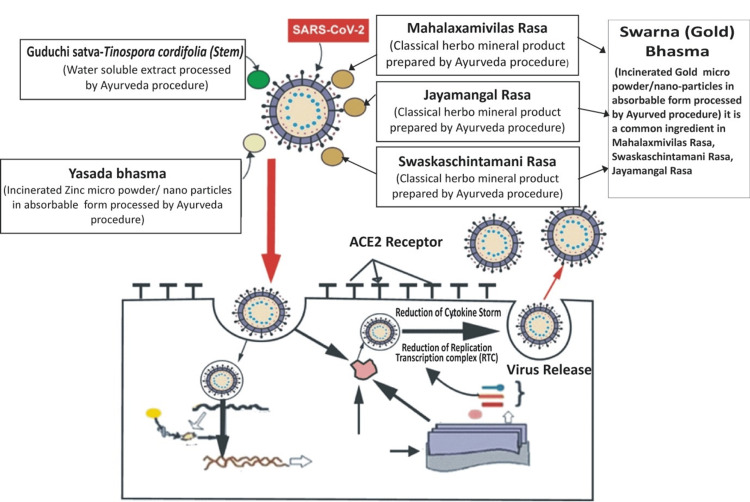
Schematic diagram showing Svarnvir-IV tablet composition and probable action on SARS-CoV-2 virus Image credit: Author Reddy

Being anti-inflammatory in nature, Yashada bhasma fights COVID-19 by reducing plasma interleukins, interferons, and TNFα levels [9]. Zinc compound inhibits viral replication through modulation of the glutathione redox system [11]. It has also been documented that zinc, along with pyrithione, inhibited the viral replication in the COVID-19 infections. Zinc efficiently subdued the multi-protein replication of RNA viruses and SARS-CoV RdRp elongation, resulting in the reduction of template binding [35]. It is also documented that the incinerated zinc nanoparticle compound, i.e., Yashada bhasma, which is a component of the Svarnvir tablet, has a mean particle size in the range of 49-80 nm [16]. The presence of zinc in the Svarnvir tablet was confirmed by SEM-EDS and ICP-MS analysis. The Svarnvir tablet showed the presence of iron, and XRD analysis revealed that iron is in the form of oxide in the Svarnvir tablet. The FDA has earlier approved iron oxide nanoparticles for the treatment of anemia, and recent docking studies have also demonstrated an efficient interaction of Fe₂O₃ nanoparticles with the spike protein of SARS-CoV-2. As a consequence of their interaction with S-RBD, iron oxide nanoparticles are thought to lead to viral inactivation by changing virus conformation [36]. Several viruses, including the H1N1 influenza virus, the human norovirus, and herpes simplex, were effectively combated by copper-based nanoparticles in their oxide, sulfide, or iodide forms [37]. Svarnvir-IV tablet composition and probable action on the SARS-CoV-2 virus are depicted in Figures 4, 5.

Western medications such as hydroxychloroquine made from cinchona bark at a concentration of EC50=0.72 μM and oseltamivir containing shikimic acid made from the spice star anise have been licensed for use in in vitro investigations utilizing SARS-CoV-2-infected Vero cells. Adhatoda vasica (AV) treatment shows the potential efficacy in decreasing the viral load in Vero cells infected with the virus of COVID-19 [38,39,40]. Svarnvir tablet showed virucidal activity against SARS-CoV-2 with an EC50 value of 0.0058 mg/ml when incubated with the SARS-CoV-2 virus for two hours. Additionally, it also demonstrated therapeutic action when administered to Vero cells that had been infected with the SARS-CoV-2 virus (0.1 MoI) for one hour, two hours, and four hours post-infection, with EC50 values of 0.094 mg/ml, 0.023 mg/ml, and 0.05 mg/ml, respectively. This demonstrates the virucidal and antiviral efficacy of the Svarnvir tablet [39].

The Svarnvir tablet treatment at various concentrations (0.05 mg/ml, 0.1 mg/ml, 0.5 mg/ml, and 1.0 mg/ml) fed to parental flies and their offspring exhibited no toxic effects on developmental progression (Figure 2A), but the number of F1 progeny (Figure 2B (I)) and cell viability slightly decrease in 0.05 mg/ml compared to 1 mg/ml Svarnvir tablet-treated wild-type (Figure 2B (III)), the parental flies were metabolically active at various doses of Svarnvir tablet as revealed by the cell viability assay (Figure 2B (II)). Therefore, biochemical assays were performed on 0.05 mg/ml (minimum dose) and 1.0 mg/ml (maximum dose) of Svarnvir tablet-treated wild-type flies, third instar larvae, and F1 adult flies. At this dosage, there were no remarkable differences observed in the biochemical parameters of parental flies and their offspring (Figure 2C (I, II, III)). Thus, the in vivo studies show that Svarnvir tablet concentrations of up to 1 mg/ml could be used as a safe formulation for flies.

 Ayurvedic formulation of the Svarnvir tablets tested to study locomotor activity rhythm and activity pattern of OregonR+Drosophila shows that the Svarnvir tablet treatment at different concentrations of 0.05 mg/ml, 0.1 mg/ml, 0.5 mg/ml, and 1.0 mg/ml fed to the wild-type strain of OregonR+ flies exhibited no significant effect on the locomotor activity rhythm and behavioral activity patterns such as bimodal pattern (crepuscular activity) (Figure 3A, 3B, 3C). The statistical analysis for activity counts/day, total activity counts, and activity counts during the light phase and dark phase does not show any significant changes when compared to the control (Figure 3D, 3E, 3G). Our study is one of the first of its kind to demonstrate the effects of the ayurvedic formulation of coronavirus on the developmental stages, cell viability, oxidative stress, circadian locomotor activity rhythm, and activity pattern of Drosophila. Overall, the study shows that different doses of the Svarnvir tablets screened up to 1.0 mg/ml concentration did not show any alteration in the circadian locomotor activity rhythm and activity patterns of Drosophila. Given the in vitro effectiveness demonstrated in the study, further investigation to establish the Svarnvir tablet as a potential antiviral regimen against SARS-CoV-2 is needed.

## Conclusions

Overall, this study highlighted that the Ayurvedic formulation of the Svarnvir tablet is a viable therapeutic approach against SARS-CoV-2 variant infections due to its safety. Developing direct-acting antivirals (DAAs) has gained momentum in recent times, and these efforts could lead to highly potent Ayurveda antiviral drugs. Based on the references to the multiplicity of infection and EC50 values obtained with experiments on Vero cells, our study showed that the Ayurvedic-formulated Svarnvir tablet has therapeutic potential by having an antiviral as well as virucidal activity against the SARS-CoV-2 virus without causing adverse effects. The combination formulation of the drug showed therapeutic potential by diminishing the relative percent cytopathic effects (CPE) inhibition in both therapeutic and virucidal modes. The maximum percent CPE inhibition of 118.3% was observed in a 0.125 mg/ml dose at two hours of challenge with SAR-CoV-2. Physiologically and morphologically healthy and active flies treated with a 1.0 mg/ml dosage further demonstrate the efficacy of the treatment. At this dosage, no alterations were found in the metabolic and biochemical activities of flies and their larvae. When compared with control flies, the treatment had no effect on circadian locomotor activity rhythms or activity patterns in Drosophila. Hence our study suggests that the usage of Svarnvir tablet up to 1.0 mg/ml concentration is safe. We achieved such a significant effect while applying a traditional medicine approach with Ayurvedic herbo-mineral combination therapy and presented both in vivo and in vitro evidence of the potential utility of Svarnvir tablet in mitigating SARS-CoV-2 with no adverse effects on physiology and morphology. Svarnvir tablet's potential in treating COVID-19 will be further explored by using the original supporting data from this study in conjunction with existing Ayurvedic traditional information. As a result, the drug can rapidly be repurposed and used to treat COVID-19 patients in a safe and highly effective manner. 
